# Supplementary material for: Distribution and abundance of azaspiracid-producing dinophyte species and their toxins in North Atlantic and North Sea waters in summer 2018
Source: PLoS One. 2020 Jun 19;15(6):e0235015. doi: 10.1371/journal.pone.0235015 (PMC7304611; doi:10.1371/journal.pone.0235015)
Supplement: S1 Table — (PDF) [file pone.0235015.s001.pdf]

S1 Table. Mass transitions m/z (Q1>Q3 mass) and their respective AZA.

Mass transition	Toxin	Collision energy (CE) [V]
716>698	AZA-33	40
796>778	AZA-33 phosphate	40
816>798	AZA-34, AZA-39	40
816>348	AZA-39	70
828>658	AZA-3	70
828>810	AZA-3, AZA-43, AZA-58	40
830>812	AZA-38, AZA-52, AZA-53	40
830>348	AZA-38, AZA-52, AZA-53	70
842>672	AZA-1	70
842>824	AZA-1, AZA-40, AZA-50	40
842>348	AZA-40	70
844>826	AZA-4, AZA-5	40
846>828	AZA-37	40
846>348	AZA-37	70
854>836	AZA-41	40
854>670	AZA-41	70
854>360	AZA-41	70
856>672	AZA-2	70
856>838	AZA-2	40
858>840	AZA-7, AZA-8, AZA-9, AZA-10, AZA-36, AZA-51	40
858>348	AZA-36, AZA-51	70
860>842	AZA-59	40
868>362	AZA-55	70
870>852	Me-AZA-2, AZA-42, AZA-54	40
870>360	AZA-42	40
872>854	AZA-11, AZA-12	40
884>866	AZA-56	40
910>892	Undescribed	40
920>804	AZA-1 phosphate, AZA-40 phosphate	40
926>908	AZA-37 phosphate	40
936>918	AZA-2 phosphate	40
938>920	AZA-36 phosphate, AZA-51 phosphate	40
940>922	AZA-59 phosphate	40
952>938	AZA-11 phosphate	40
