# Supplementary material for: Distribution and abundance of azaspiracid-producing dinophyte species and their toxins in North Atlantic and North Sea waters in summer 2018
Source: PLoS One. 2020 Jun 19;15(6):e0235015. doi: 10.1371/journal.pone.0235015 (PMC7304611; doi:10.1371/journal.pone.0235015)
Supplement: S2 Table — n.d. = not detected; n.a. = no data available. (PDF) [file pone.0235015.s002.pdf]

Distribution and abundance of azaspiracid-producing dinophyte species and their toxins in North Atlantic and North Sea waters in summer 2018

S. Wietkamp, B. Krock, D. Clarke, D. Voß, R. Salas, J. Kilcoyne & U. Tillmann

S2 Table. Species counts (cells L^{-1}) based on qPCR (Cell number calculation based on mean Ct values of three technical replicates) and microscopy, as well as toxin amount (pg L^{-1}) based on LC-MS/MS for each station. n.d. = not detected; n.a. = no data available.

Station	Lat [°N]	Long [°E]	*Az. spinosum* [cells L^{-1}]	*Az. poporum* [cells L^{-1}]	*Am. languida* [cells L^{-1}]	qPCR [sum cells L^{-1}]	Microscopy [sum cells L^{-1}]	AZA-1 [pg L^{-1}]	AZA-2 [pg L^{-1}]	AZA-33 [pg L^{-1}]	AZA-38 [pg L^{-1}]	AZA-39 [pg L^{-1}]
**St.1**	53.765	6.239	n.d.	n.d.	n.d.	n.d.	n.a.	n.d.	n.d.	n.d.	n.d.	n.d.
**St.2**	53.119	4.332	n.d.	n.d.	n.d.	n.d.	n.a.	n.d.	n.d.	n.d.	n.d.	n.d.
**St.3**	52.668	2.246	n.d.	n.d.	n.d.	n.d.	n.a.	n.d.	n.d.	n.d.	n.d.	n.d.
**St.4**	51.509	2.680	n.d.	n.d.	n.d.	n.d.	n.a.	n.d.	n.d.	n.d.	n.d.	n.d.
**St.5**	50.807	0.955	n.d.	19	n.d.	19	n.a.	n.d.	n.d.	n.d.	n.d.	n.d.
**St.6**	50.239	-0.948	n.d.	n.d.	n.d.	n.d.	n.a.	n.d.	n.d.	n.d.	n.d.	n.d.
**St.7**	49.772	-2.833	n.d.	57	n.d.	57	n.a.	n.d.	n.d.	n.d.	n.d.	n.d.
**St.8**	48.913	-4.899	n.d.	1	312	313	n.a.	n.d.	n.d.	n.d.	n.d.	n.d.
**St.9**	50.047	-5.967	509	n.d.	314	823	n.a.	17	22	3	n.d.	n.d.
**St.10**	50.341	-6.069	1,860	1	82	1,942	n.a.	56	24	8	n.d.	n.d.
**St.11**	50.636	-6.172	692	n.d.	931	1,623	n.a.	15	n.d.	n.d.	n.d.	n.d.
**St.12**	50.926	-6.266	2,375	n.d.	n.d.	2,375	n.a.	16	n.d.	n.d.	n.d.	n.d.
**St.13**	51.220	-6.364	785	24	700	1,509	n.a.	18	n.d.	n.d.	n.d.	n.d.
**St.14**	51.515	-6.480	59	7	3	68	n.a.	n.d.	n.d.	n.d.	n.d.	n.d.
**St.15**	51.804	-6.582	151	34	10	195	n.a.	7	n.d.	n.d.	n.d.	n.d.
**St.16**	52.099	-6.709	n.d.	2	4	6	n.a.	n.d.	n.d.	n.d.	n.d.	n.d.
**St.17**	51.908	-7.452	88	29	n.d.	117	n.a.	n.d.	n.d.	n.d.	n.d.	n.d.
**St.18**	51.708	-8.233	n.d.	n.d.	15	15	n.a.	n.d.	n.d.	n.d.	n.d.	n.d.
**St.19**	50.520	-9.070	n.d.	n.d.	7	7	n.a.	n.d.	n.d.	n.d.	n.d.	n.d.
**St.20**	50.683	-9.071	n.d.	n.d.	7	7	n.a.	n.d.	n.d.	n.d.	n.d.	n.d.
**St.21**	50.850	-9.066	1,119	7	956	2,082	n.a.	n.d.	n.d.	n.d.	n.d.	n.d.
**St.22**	51.021	-9.068	1,354	n.d.	10	1,364	n.a.	n.d.	5	n.d.	n.d.	n.d.
**St.23**	51.185	-9.069	7	5	18	30	n.a.	13	11	n.d.	n.d.	n.d.
**St.24**	51.349	-9.060	n.d.	n.d.	1	1	n.a.	12	n.d.	n.d.	n.d.	n.d.
**St.25**	51.517	-9.071	668	n.d.	188	857	n.a.	9	n.d.	n.d.	n.d.	n.d.
**St.26**	51.388	-9.318	n.d.	n.d.	1,489	1,489	n.a.	n.d.	n.d.	n.d.	n.d.	n.d.
**St.27**	51.381	-9.696	178	14	7	199	n.a.	12	n.d.	n.d.	n.d.	n.d.
**St.28**	51.132	-11.150	n.d.	1	4	5	100	n.d.	n.d.	n.d.	n.d.	n.d.
**St.29**	51.235	-10.884	n.d.	n.d.	5	5	100	n.d.	n.d.	n.d.	n.d.	n.d.
**St.30**	51.334	-10.574	2,953	n.d.	n.d.	2,953	100	27	30	n.d.	n.d.	n.d.
**St.31**	51.639	-9.715	34,273	n.d.	58	34,331	6,500	59	44	16	n.d.	n.d.
**St.32**	51.542	-10.010	3,889	4	409	4,301	500	85	29	17	n.d.	n.d.

**St.33**	51.437	-10.288	1,660	n.d.	123	1,783	500	13	n.d.	n.d.	n.d.	n.d.
**St.34**	51.737	-10.519	13,710	1	291	14,003	2,500	129	66	33	n.d.	n.d.
**St.35**	52.029	-10.777	6,061	n.d.	1,486	7,547	n.a.	53	n.d.	9	n.d.	n.d.
**St.36**	52.289	-10.410	10,904	n.d.	33	10,937	n.a.	78	42	37	n.d.	n.d.
**St.37**	53.080	-9.414	7,815	n.d.	1	7,816	7,000	89	68	25	n.d.	n.d.
**St.38**	52.793	-9.688	3,818	n.d.	n.d.	3,818	2,800	41	20	9	n.d.	n.d.
**St.39**	52.541	-10.049	9,491	n.d.	n.d.	9,491	n.a.	137	50	32	n.d.	n.d.
**St.40**	53.777	-11.556	n.d.	n.d.	n.d.	n.d.	100	n.d.	n.d.	n.d.	n.d.	n.d.
**St.41**	53.746	-11.221	n.d.	n.d.	3	3	50	n.d.	n.d.	n.d.	n.d.	n.d.
**St.42**	53.715	-10.891	3,630	n.d.	106	3,736	700	30	2	9	n.d.	n.d.
**St.43**	53.684	-10.561	6,907	n.d.	53	6,960	1,500	95	46	14	n.d.	n.d.
**St.44**	53.656	-10.224	5,689	n.d.	n.d.	5,690	3,000	676	305	166	n.d.	n.d.
**St.45**	53.629	-9.906	83,059	n.d.	n.d.	83,059	18,000	745	325	204	n.d.	n.d.
**St.46**	53.935	-10.342	5,612	n.d.	n.d.	5,613	1,300	65	38	21	n.d.	n.d.
**St.47**	54.336	-10.114	26,301	n.d.	n.d.	26,301	9,000	102	41	18	n.d.	n.d.
**St.48**	54.357	-9.397	4,063	n.d.	57	4,120	1,200	46	33	17	n.d.	n.d.
**St.49**	55.405	-10.773	n.d.	n.d.	n.d.	n.d.	50	n.d.	n.d.	n.d.	n.d.	n.d.
**St.50**	55.214	-10.288	n.d.	n.d.	n.d.	n.d.	50	n.d.	n.d.	n.d.	n.d.	n.d.
**St.51**	55.028	-9.811	n.d.	n.d.	n.d.	n.d.	n.d.	n.d.	n.d.	n.d.	n.d.	n.d.
**St.52**	54.557	-8.300	n.d.	n.d.	n.d.	n.d.	20	n.d.	n.d.	n.d.	n.d.	n.d.
**St.53**	54.645	-8.874	16,429	n.d.	3	16,432	6,000	113	54	45	n.d.	n.d.
**St.54**	54.839	-9.324	10,245	n.d.	2	10,246	4,000	62	39	17	n.d.	n.d.
**St.55**	56.107	-8.594	4	n.d.	n.d.	4	50	n.d.	n.d.	n.d.	n.d.	n.d.
**St.56**	56.510	-8.399	n.d.	n.d.	14	14	50	n.d.	n.d.	n.d.	n.d.	n.d.
**St.57**	56.916	-8.245	n.d.	n.d.	n.d.	n.d.	n.d.	n.d.	n.d.	n.d.	n.d.	n.d.
**St.58**	57.751	-8.201	n.d.	n.d.	n.d.	n.d.	n.d.	n.d.	n.d.	n.d.	n.d.	n.d.
**St.59**	58.141	-7.948	n.d.	n.d.	1	1	n.d.	n.d.	n.d.	n.d.	n.d.	n.d.
**St.60**	58.475	-7.470	n.d.	n.d.	n.d.	n.d.	n.d.	n.d.	n.d.	n.d.	n.d.	n.d.
**St.61**	58.737	-4.336	n.d.	n.d.	n.d.	n.d.	n.d.	n.d.	n.d.	n.d.	n.d.	n.d.
**St.62**	58.729	-3.394	n.d.	n.d.	n.d.	n.d.	n.d.	n.d.	n.d.	n.d.	n.d.	n.d.
**St.63**	58.495	-2.529	n.d.	n.d.	6	6	20	n.d.	n.d.	n.d.	n.d.	n.d.
**St.64**	57.992	-1.275	n.d.	1	n.d.	1	100	n.d.	n.d.	n.d.	n.d.	n.d.
**St.65**	57.690	-0.530	n.d.	1	n.d.	1	n.d.	n.d.	n.d.	n.d.	n.d.	n.d.
**St.66**	57.384	0.214	n.d.	n.d.	n.d.	n.d.	n.d.	n.d.	n.d.	n.d.	n.d.	n.d.
**St.67**	56.890	1.436	5	n.d.	n.d.	5	n.d.	n.d.	n.d.	n.d.	n.d.	n.d.
**St.68**	56.590	2.167	19	n.d.	276	295	50	n.d.	n.d.	n.d.	n.d.	n.d.
**St.69**	56.288	2.880	102	5	n.d.	107	500	n.d.	n.d.	n.d.	n.d.	n.d.
**St.70**	55.739	4.009	1,074	n.d.	n.d.	1,074	1,300	10	n.d.	n.d.	n.d.	n.d.
**St.71**	55.360	4.651	6,198	n.d.	122,810	129,009	275,000	15	n.d.	n.d.	234	384
**St.72**	55.176	5.470	n.d.	n.d.	802	802	7,700	n.d.	n.d.	n.d.	81	74
**St.73**	54.673	6.621	n.d.	n.d.	656	656	4,400	n.d.	n.d.	n.d.	n.d.	n.d.
**St.74**	54.369	7.311	n.d.	n.d.	5,592	5,592	2,600	n.d.	n.d.	n.d.	n.d.	n.d.
**St.75**	54.069	7.990	n.d.	n.d.	69	69	50	n.d.	n.d.	n.d.	n.d.	n.d.
