# Supplementary figures and images for: Distribution and abundance of azaspiracid-producing dinophyte species and their toxins in North Atlantic and North Sea waters in summer 2018
Source: PLoS One. 2020 Jun 19;15(6):e0235015. doi: 10.1371/journal.pone.0235015 (PMC7304611; doi:10.1371/journal.pone.0235015)

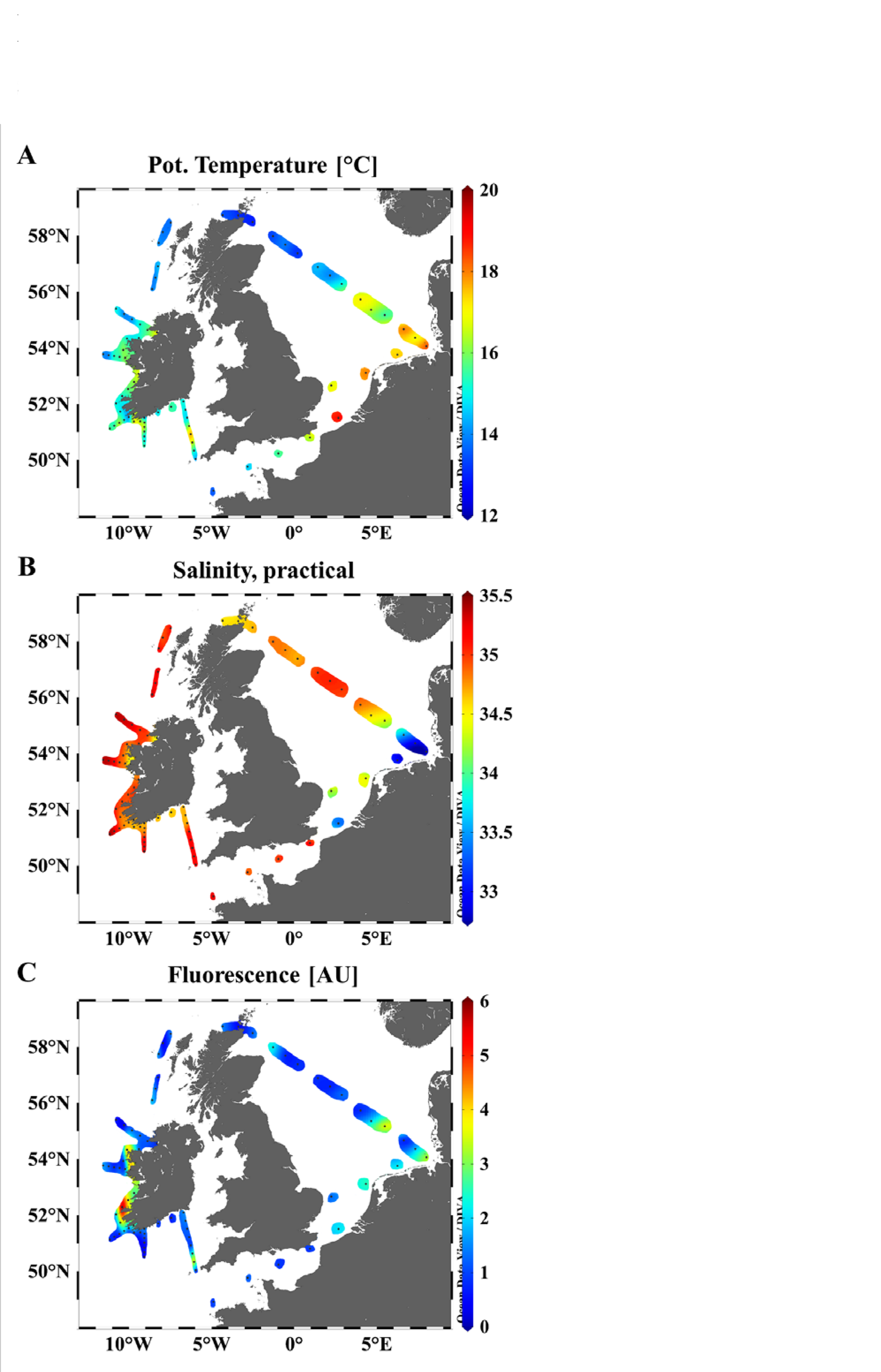

Supplement: S1 Fig — Geographical distribution of temperature (A), salinity (B) and fluorescence (C) averaged for the upper layer defined as the maximum sampling depth at each station. (TIF) [file pone.0235015.s003.tif]

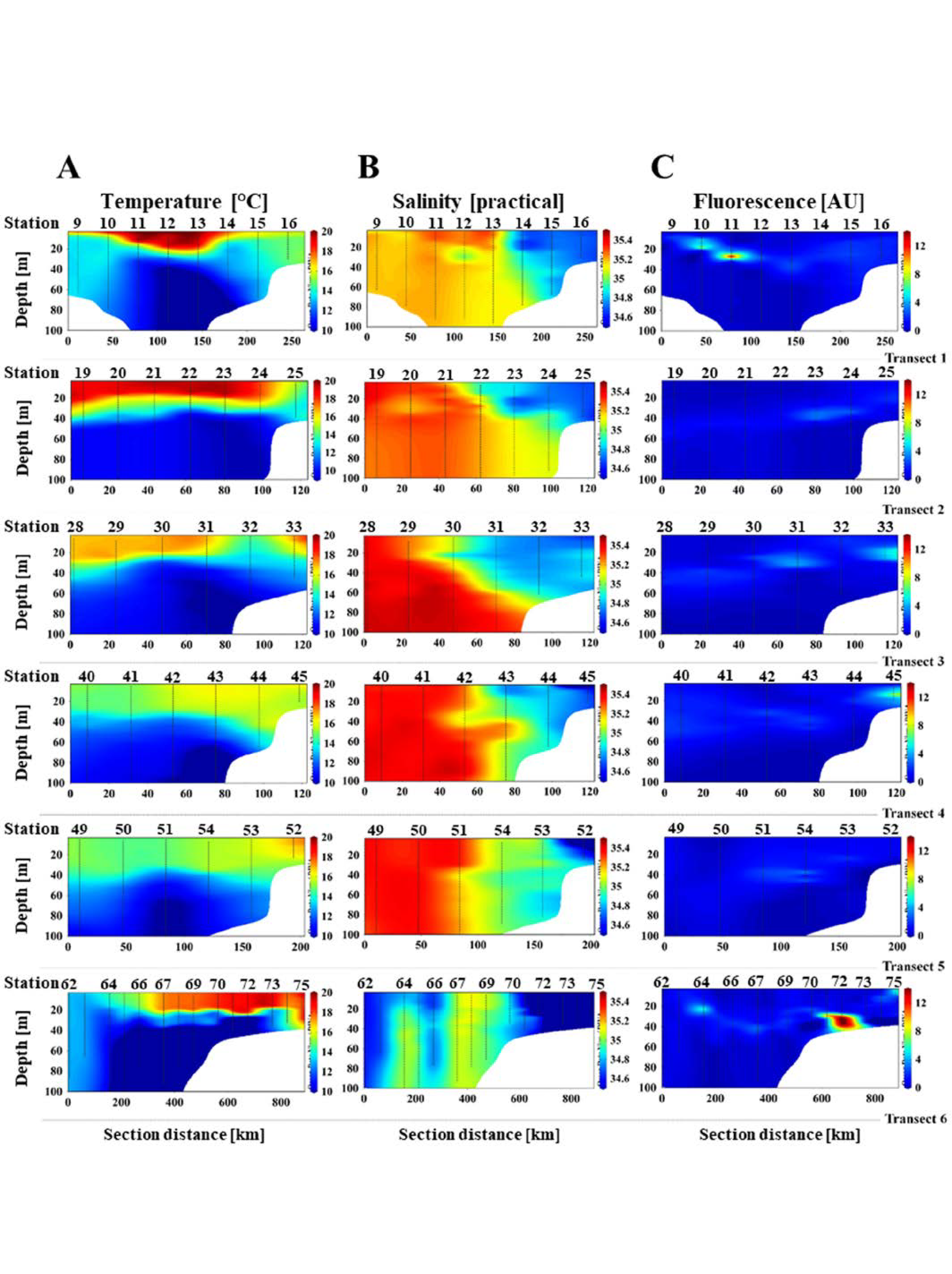

Supplement: S2 Fig — Depth profiles of temperature (A), salinity (B) and fluorescence (C) for the six defined sampling transects T1 –T6 as indicated in Fig 1B. (TIF) [file pone.0235015.s004.tif]
